# Ancestral population reconstitution from isofemale lines as a tool for experimental evolution

**DOI:** 10.1002/ece3.2402

**Published:** 2016-08-30

**Authors:** Pierre Nouhaud, Ray Tobler, Viola Nolte, Christian Schlötterer

**Affiliations:** ^1^ Institut für Populationsgenetik Vetmeduni Vienna Vienna Austria; ^2^ Present address: Ray Tobler, Australian Centre for Ancient DNA School of Biological Sciences University of Adelaide Adelaide SA Australia

**Keywords:** Adaptation, evolve and resequence, experimental evolution, isofemale line, whole‐genome sequencing

## Abstract

Experimental evolution is a powerful tool to study adaptation under controlled conditions. Laboratory natural selection experiments mimic adaptation in the wild with better‐adapted genotypes having more offspring. Because the selected traits are frequently not known, adaptation is typically measured as fitness increase by comparing evolved populations against an unselected reference population maintained in a laboratory environment. With adaptation to the laboratory conditions and genetic drift, however, it is not clear to what extent such comparisons provide unbiased estimates of adaptation. Alternatively, ancestral variation could be preserved in isofemale lines that can be combined to reconstitute the ancestral population. Here, we assess the impact of selection on alleles segregating in newly established *Drosophila* isofemale lines. We reconstituted two populations from isofemale lines and compared them to two original ancestral populations (AP) founded from the same lines shortly after collection. No significant allele frequency changes could be detected between both AP and simulations showed that drift had a low impact compared to Pool‐Seq‐associated sampling effects. We conclude that laboratory selection on segregating variation in isofemale lines is too weak to have detectable effects, which validates ancestral population reconstitution from isofemale lines as an unbiased approach for measuring adaptation in evolved populations.

## Introduction

Combining experimental evolution and high‐throughput sequencing is a powerful approach to study adaptation (Barrick and Lenski 2013; Achaz et al. 2014; Long et al. 2015; Schlötterer et al. 2015). By monitoring allele frequency changes (AFCs) in populations exposed to new environments, evolve and resequence approaches (E&R, Turner et al. 2011) permit the identification of targets of selection and provide new insights into fundamental evolutionary questions such as the genomic architecture of adaptive traits (Burke et al. 2010; Parts et al. 2011; Orozco‐terWengel et al. 2012; Remolina et al. 2012), the reproducibility of evolution (Teotónio et al. 2009; Chan et al. 2012; Tenaillon et al. 2012; Burke et al. 2014), or the tempo of evolutionary change (Barrick and Lenski 2013). While the most common experimental evolution design relies on microbes with large population sizes to study the impact of novel mutations occurring during the experiment (reviewed in Barrick and Lenski 2013), studying adaptation from standing variation in outcrossing systems is enjoying increasing popularity. In particular, the genus *Drosophila*, a classical workhorse for experimental evolution in outcrossing organisms (Harshman and Hoffmann 2000; Burke and Rose 2009) – with studies sometimes covering many generations (e.g., Rose 1984; Rose et al. 1992; Chippindale et al. 1994, 1998; Teotonio et al. 2002; Matos et al. 2004; Simões et al. 2008) – is also commonly used in E&R studies (e.g., Burke et al. 2010; Turner et al. 2011; Orozco‐terWengel et al. 2012; Tobler et al. 2014; Franssen et al. 2015; Kang et al. 2016).

One particular challenge for experimental evolution studies analyzing adaptation from standing variation is to quantify the adaptive response caused by the novel selective regime. The classic approach for experimental evolution in *Drosophila* is to contrast evolved populations against an outbred reference population (hereafter referred as reference population), which had adapted to the laboratory conditions for many generations (e.g., Rose et al. 1992; Chippindale et al. 1998; Teotonio et al. 2002; Matos et al. 2004; Simões et al. 2008; Burke et al. 2010; Turner et al. 2011; Jha et al. 2015). Assuming that the reference population is not evolving during the experiment, this design permits the distinction between a generic adaptive response to the laboratory and adaptation to the experimental conditions of interest. For E&R studies in particular, this design faces some challenges. Apart from genetic drift changing allele frequencies during the maintenance of the reference population, the relatively small population size of the reference population increases the haplotype structure in the (evolved) reference population (Kofler and Schlötterer 2014). Because E&R studies are strongly affected by linkage disequilibrium in the ancestral population (Kofler and Schlötterer 2014), Tobler et al. (2015) proposed an alternative strategy. Isofemale lines act to preserve the variation in the ancestral population (AP) and allow for a reconstituted ancestral population (RAP) to be generated at any time during the experiment. Compared to using a reference population, the advantage of reconstituting the ancestral population from isofemale lines is that the level of variation and linkage disequilibrium remains very similar over time allowing to establish almost identical RAPs at multiple time points throughout the experiment. Instead of using reference populations, E&R studies can be initiated from freshly established isofemale lines (Orozco‐terWengel et al. 2012; Tobler et al. 2014; Franssen et al. 2015). One potential complication of using RAPs arises when freshly established isofemale lines are being used to start the experimental evolution study: Variation segregating within the isofemale lines provides the potential for an adaptive response during the maintenance of these lines. This caveat could be avoided by inbreeding the isofemale lines for 10–20 generations. On the other hand, because isofemale lines are typically maintained under small population sizes in the laboratory, it seems likely that most of the variants are effectively neutral (*N*_e_s < 1, Haldane 1927).

Because fresh isofemale lines contribute more haplotype diversity to the founder population of an E&R study than a RAP founded from the same isofemale lines after several generations of inbreeding, it is important to know whether allele frequencies in the isofemale lines are systematically shifted during their maintenance. Such a result would compromise the utility of reconstituted ancestral populations as suitable means to measure the changes in the evolved populations at later stages of the experiment. Here, we address this question by comparing allele frequencies from APs of *Drosophila melanogaster* and *Drosophila simulans* derived from freshly established isofemale lines to those from RAPs established from isofemale lines maintained for several additional years. With no SNP being significantly differentiated between either of the ancestral and RAP pairs, we conclude that selection in isofemale lines is not efficient, making RAPs a powerful approach to measure phenotypic changes during experimental evolution – even when freshly established isofemale lines are being used.

## Methods

### Experimental populations

The ancestral *D. melanogaster* populations used in this study correspond to two of the three populations described in Orozco‐terWengel et al. (2012) as base (B) populations. They were generated from 113 isofemale lines that were established from wild *D. melanogaster* females sampled in northern Portugal in July 2008 (Orozco‐terWengel et al. 2012). After five generations in the laboratory, five females from each isofemale line were combined to create ten replicated populations (i.e., 565 females per replicate), two of which were used for this study and will be hereafter referred to as AP. The isofemale lines were subsequently maintained at low to moderate densities (approximately 20–100 individuals/vial/generation) and under laboratory conditions (17–20°C, without tightly controlled light–dark conditions). Following the procedure described in Tobler et al. (2015), we created two reconstituted ancestral populations (RAPs) in early 2015 (after approximately 100 generations laboratory maintenance) by combining one female from each of 110 remaining isofemale lines (of originally 113) into each RAP.

The *D. simulans* populations derive from 202 isofemale lines collected by R. Tobler in Tallahassee, Florida (USA), in November 2011. They were maintained under the same conditions as the *D. melanogaster* lines, and the APs were set up after nine generations in the laboratory by combining five females from each isofemale line into each replicate. The *D. simulans* RAPs were generated at the same time as those for *D. melanogaster* (i.e., after approximately 50 generations laboratory maintenance) using a single female of each of the 202 lines per replicate.

### Genome sequencing

Whole‐genome sequencing was performed for two APs and two RAPs using either Illumina HiSeq2000 with 100‐bp paired‐end reads (*D. melanogaster* APs and RAPs, *D. simulans* APs) or Illumina HiSeq2500 with 125‐bp paired‐end reads (*D. simulans* RAPs). Genomic DNA was extracted from all pooled females per replicate (*D. melanogaster* APs: 565 females, *D. melanogaster* RAPs: 110 females, *D. simulans* APs: 1010 females, *D. simulans* RAPs: 202 females) to be sequenced as a single sample (i.e., Pool‐Seq, Futschik and Schlötterer 2010).

For *D. melanogaster* APs, new libraries were prepared from the DNA extractions described in Orozco‐terWengel et al. (2012). Starting from 1.5 μg genomic DNA, paired‐end libraries were generated using the NEBNext^®^ DNA Library Prep Master Mix Set reagents (E6040L) with TruSeq index adapters. An initial size selection was performed before PCR using AMPureXP beads (Beckman Coulter, Brea, CA). PCR was carried out for 10 cycles at 60°C annealing temperature with the TruSeq PCR Mastermix. The insert size distribution of the final libraries was tightened to a mean of approximately 280 bp by size selection on an agarose gel.

For the *D. melanogaster* RAPs, libraries were generated from 4 μg genomic DNA again using NEBNext Mastermix reagents followed by bead‐based size selection for an insert size of 330 bp. PCR was carried out using Phusion polymerase with six cycles at 65°C and NEBNext single index barcodes.

Libraries for the *D. simulans* APs were prepared from 2.5 μg genomic DNA using the TruSeq DNA PCR‐free library preparation protocol (Illumina Inc., San Diego, CA) with size selection for 420‐bp fragment size and single index adapters.

The *D. simulans* RAP libraries were generated from 5 μg genomic DNA following the same protocol (Illumina Inc.), but with size selection for 650‐bp fragment size and dual index adapters.

### Mapping and variant calling

Raw reads were trimmed using PoPoolation 1.2.2 (Kofler et al. 2011a) and mapped against the *D. melanogaster* (version 6.03) or *D. simulans* M252 (version 1.1, Palmieri et al. 2015) reference genomes along with genomes from *Wolbachia pipientis* (AE017196.1), *Acetobacter pasteurianus* (AP011170.1), and *Lactobacillus brevis* (CP000416.1) using BWA 0.6.2 (Li and Durbin 2009) with the mapping parameters: ‐o 1 ‐n 0.01 ‐l 200 ‐e 12 ‐d 12. Duplicates were removed from the SAM files with PICARDTOOLS (http://broadinstitute.github.io/picard, last accessed on 03/23/2016), and reads mapped in proper pairs or with a mapping quality superior to 20 were filtered using SAMTOOLS 1.2 (Li et al. 2009). After conversion to mpileup format, repeated sequences were identified using REPEATMASKER 4.0.6 (Smit et al. 2013) and were masked along with indels and their 5‐bp flanking sequences. The mpileup file was converted to a synchronized file (Kofler et al. 2011a) used to call variants on the major chromosome arms (i.e., X, 2L, 2R, 3L, 3R, 4) and obtain allele counts for each population. Only sites displaying a minimum base quality of 30, coverage between 30 and 500× for each population and a minimum allele count of eight across all populations (to avoid putative sequencing errors) were considered.

### Allele frequency change analyses

Identification of consistent AFCs between pairs of AP‐RAP populations was performed using the Cochran–Mantel–Haenszel test (CMH, Agresti 2002), which is well suited for candidate inference in E&R studies (Kofler and Schlötterer 2014). Allele counts were subsampled without replacement to a target coverage of 60× at each genomic position for *D. melanogaster* and 30× for *D. simulans*, accounting for the population sample with lowest coverage (Table 1). CMH tests were performed for each SNP using PoPoolation 2 (Kofler et al. 2011b) to compare two pairs of populations (AP1 vs. RAP1; AP2 vs. RAP2) within each species. Differences in insert sizes (Table 1) may cause artifactual AFCs due to mapping problems. We therefore used an additional mapper and intersected the results to reduce these artifacts (R. Kofler, A. M. Langmüller, P. Nouhaud, K. A. Otte, C. Schlötterer, in press). The second mapper was NOVOALIGN (Novocraft 2010) with the mapping parameters: ‐o SAM ‐o FullNW ‐r Random. The same pipeline presented above was applied on the resulting BAM files. SNPs identified with both mappers were retained along with their maximal CMH test *P*‐value, with a SNP‐wise Benjamini–Hochberg procedure being applied to correct for multiple testing.

**Table 1:** Overview of the data. In *Drosophila melanogaster* and *Drosophila simulans*, ancestral (AP) and reconstituted ancestral populations (RAP) were sequenced in two replicates. Sequencing statistics include coverage computed from BAM files obtained through BWA using SAMTOOLS, mean insert size length, and standard deviation (SD) inferred by PICARDTOOLS

Species	Population	No. of isofemale lines	Coverage	Insert size length (SD)
*D. melanogaster*	AP1	113	103	289 (71.4)
	AP2	113	134	283 (75.1)
	RAP1	110	61.2	328 (85.6)
	RAP2	110	79.1	346 (87.7)
*D. simulans*	AP1	202	154	374 (66.1)
	AP2	202	168	386 (67.4)
	RAP1	202	39.0	524 (140)
	RAP2	202	69.8	522 (139)

### Drift simulations

We performed simulations based on *D. simulans* data to assess the relative importance of genetic drift and sequencing coverage on the AFCs. For these simulations, each of the 202 isofemale lines were assumed to derive from a natural parental population at Hardy–Weinberg equilibrium and to result from a cross between two individuals from this parental population (hence each carried 4N segregating chromosomes). A sample of 5,000,000 SNPs was randomly drawn from this parental population, assuming independence between alleles and an exponentially distributed allele frequency spectrum in the parental population. The number of homozygotes and heterozygotes was assumed to be trinomially distributed for each locus with expected probabilities deriving from the Hardy–Weinberg equilibrium (i.e., *p* ^2^, *q* ^2^, 2*p*q). Trinomial sampling was performed twice for each isofemale line to take into account the 4N segregating chromosomes. For each resulting heterozygote isofemale line, drift was then simulated assuming that the process would result in the fixation of one or the other allele. Thus, the probability of fixation was binomially distributed, with *p* of 0.25, 0.5, or 0.75 (i.e., the allele was on 1–3 chromosomes) and the number of heterozygotes *n* varying accordingly. This expectation of fixation seems reasonable as isofemale lines were maintained under a small census population size for at least 3 years (3‐week generation time implies that this amounts to at least 50 generations). AFCs were computed by subtracting the initial from the final allele frequencies after drift. A round of binomial sampling was also applied on both initial and final allele counts to account for the effect of Pool‐Seq with coverage drawn from the empirical coverage distributions of *D. simulans* and the AFCs recalculated from these corrected values. Quantile–quantile plots were realized between empirical and simulated datasets to assess the fit of simulations (Fig. S1).

Unless stated otherwise, all data analysis was performed under R version 3.2.2 (R Core Team 2015).

## Results

**Figure 1:**
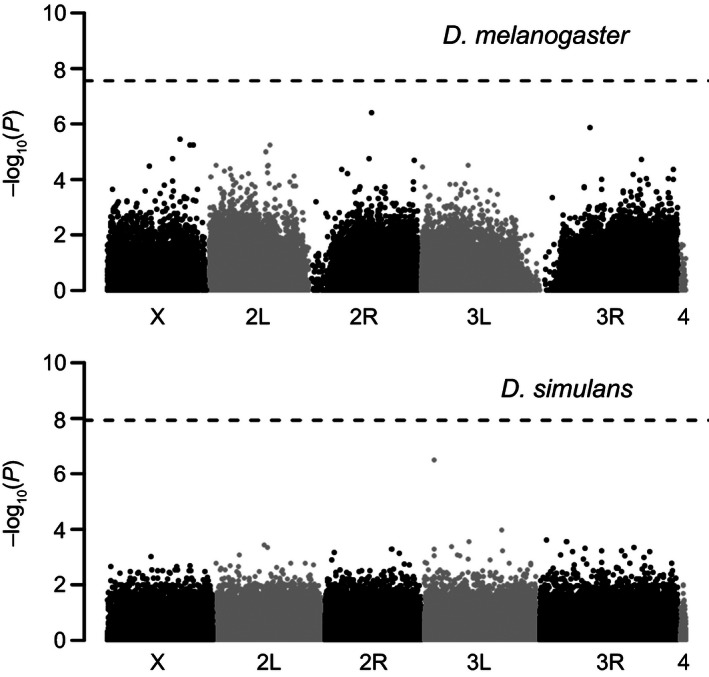
Genomic distribution of allele frequency differences inferred for *Drosophila melanogaster* (upper panel) and *Drosophila simulans* (lower panel). The negative log10‐transformed *P*‐values obtained for each SNP from a Cochran–Mantel–Haenszel test are displayed together with their chromosomal position. No SNPs were detected above the Benjamini–Hochberg FDR threshold of 0.01 (depicted as a dashed line) in either species comparison. The same results were obtained after filtering for SNPs with a minor allele frequency ≥0.2 before applying FDR correction.

We studied the impact of selection on alleles segregating in freshly established *D. melanogaster* and *D. simulans* isofemale lines. To do so, we used Pool‐Seq to compare allele frequency estimates from APs established from freshly collected isofemale lines to populations reconstituted from the same isofemale lines after several years of maintenance in the laboratory (RAPs). Trimmed reads were mapped with two different algorithms to get rid of potential artifacts induced by the differences in insert size length between the APs and RAPs (Table 1). This dual calling strategy led to the identification of ~362,000 and ~860,000 high‐quality SNPs for *D. melanogaster* and *D. simulans*, respectively. We attribute the lower number of SNPs in *D. melanogaster* to coverage fluctuations, which differ between the libraries of AP and RAP due to the modifications in library preparation protocols for RAPs (data not shown). Applying CMH tests for significant differences in allele frequencies between the two groups of populations in both species, we did not identify any SNP displaying a FDR < 0.01 (Fig. 1). Additionally, no enrichment of SNPs with pronounced frequency change was observed when compared to neutral simulations (Fig. S2). These results suggest that selection is too weak in isofemale lines to result in significant allele frequency differences between APs and RAPs.

**Figure 2:**
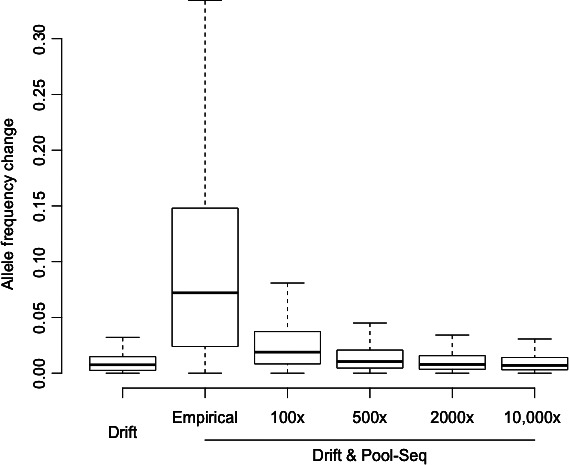
Relative impacts of drift and Pool‐Seq‐associated sampling. Neutral simulations assuming 202 isofemale lines show that allele frequency change (AFC) due to genetic drift only (drift) is expected to be weak, whereas Pool‐Seq (modeled as a round of binomial sampling on allele counts after drift) is responsible for an inflation of AFC that decreases with higher coverage values. Empirical AFC is computed using coverage values from the *Drosophila simulans* populations (see Table 1).

To shed further light into the influence of segregating variation in the isofemale lines, we studied the relative effects of drift and random sampling of chromosome fragments during Pool‐Seq on the observed AFCs. We simulated the evolution of allele frequencies for 5 million SNPs segregating in 202 isofemale lines and accounted for Pool‐Seq sampling on the initial and final allele frequencies. When assuming genetic drift only, very small differences were observed between APs and RAPs (median AFC: 0.0074, Fig. 2). However, approximating Pool‐Seq through a round of binomial sampling drastically increased AFCs, which only decreased with coverage values far beyond the reach of most research projects (Fig. 2).

## Discussion

Experimental evolution is a pertinent strategy to gain insights into fundamental evolutionary questions (Kawecki et al. 2012). In *Drosophila*, experiments usually start from a laboratory‐adapted reference population that is subject to drift even after the selection experiment has started (e.g., Rose et al. 1992; Chippindale et al. 1998; Teotonio et al. 2002; Matos et al. 2004; Simões et al. 2008; Burke et al. 2010; Turner et al. 2011; Jha et al. 2015). The relatively small population size increases haplotype structure in the reference population – and consequently in the ancestral population, thus impeding the identification of putative targets of selection by E&R (Kofler and Schlötterer 2014). Alternatively, the ancestral population could be established from isofemale lines (Tobler et al. 2015), which preserves the linkage disequilibrium of the natural source population and allows the reconstitution of ancestral populations when needed. However, because freshly established isofemale lines maintain segregating variation even after a few generations in the laboratory (Endler et al. 2016), they may be subject to an adaptive response in the laboratory until they are fully inbred. We tested for such adaptive responses by comparing populations established from freshly established lines to populations reconstituted from the same isofemale lines after at least 3 years of inbreeding.

Genome‐wide analyses of both *D. melanogaster* and *D. simulans* did not reveal a significant AFC between APs and RAPs, suggesting that laboratory adaptation and drift did not significantly impact the evolution of allele frequencies. This observation is not surprising because the isofemale lines have been maintained at low to moderate densities (approximately 20–100 individuals/vial/generation) whereby most segregating variants are expected to be effectively neutral (N_e_s < 1, Haldane 1927). Moreover, our simulations suggest that most of the AFCs measured between APs and RAPs can be attributed to the stochasticity associated with Pool‐Seq, while drift only accounts for a small amount of change.

The reconstitution of ancestral populations from isofemale lines provides several benefits for measuring adaptation in experimental evolution studies, from avoiding inbreeding associated with the maintenance of a reference population to preserving a high haplotype diversity in the population, facilitating the identification of targets of selection (Kofler and Schlötterer 2014). To conclude, in this study we showed that E&R studies using *Drosophila* can be initiated from freshly collected isofemale lines with no significant AFCs in RAPs even in cases when isofemale lines have been maintained for more than 6 years.

Freshly established isofemale lines have the advantage of multiple founder genotypes for each isofemale line, which is expected to result in a better resolution of an E&R study (Kofler and Schlötterer 2014). Provided that a sufficiently large number of inbred isofemale lines are available to assure a good mapping resolution, it may be preferable to start an E&R study from them because RAPs will be identical to the founder population. Note, however, that even when all DGRP lines would be used, this corresponds to <60 freshly collected isofemale lines, which is at the low end of what is currently being used in many *Drosophila* E&R studies (e.g., Tobler et al. 2014; Kellermann et al. 2015). Hence, given the moderate size of available inbred isofemale line collections and the considerable effort and time to establish them, we anticipate that freshly collected isofemale lines will continue to be used to seed E&R studies. RAPs from these isofemale lines will provide an unbiased reference population for phenotyping the evolved populations, allowing to better document their adaptation.

## Conflict of interest

None declared.

## Data Accessibility

The FASTQ files for all populations analyzed in the study are available from the European Nucleotide Archive (ENA, http://www.ebi.ac.uk/ena/data/view/PRJEB15225). Synchronized mpileup files and scripts used for simulations are available from the Dryad Digital Repository (http://dx.doi.org/10.5061/dryad.ks01r).

## Supporting information


**Figure S1.** Quantile‐Quantile plot of simulated versus empirical allele frequency changes (AFC) for *D. melanogaster*.
**Figure S2.** Distribution of simulated and empirical allele frequency changes (AFC) for *D. simulans*.
