# Supplementary material for: Ancestral population reconstitution from isofemale lines as a tool for experimental evolution
Source: Ecol Evol. 2016 Aug 30;6(20):7169–75. doi: 10.1002/ece3.2402 (PMC5114691; doi:10.1002/ece3.2402)
Supplement: Supplementary file 1 — Figure S1. Quantile‐Quantile plot of simulated versus empirical allele frequency changes (AFC) for D. melanogaster. Figure S2. Distribution of simulated and empirical allele frequency changes (AFC) for D. simulans. [file ECE3-6-7169-s001.pdf]

Supplementary Material

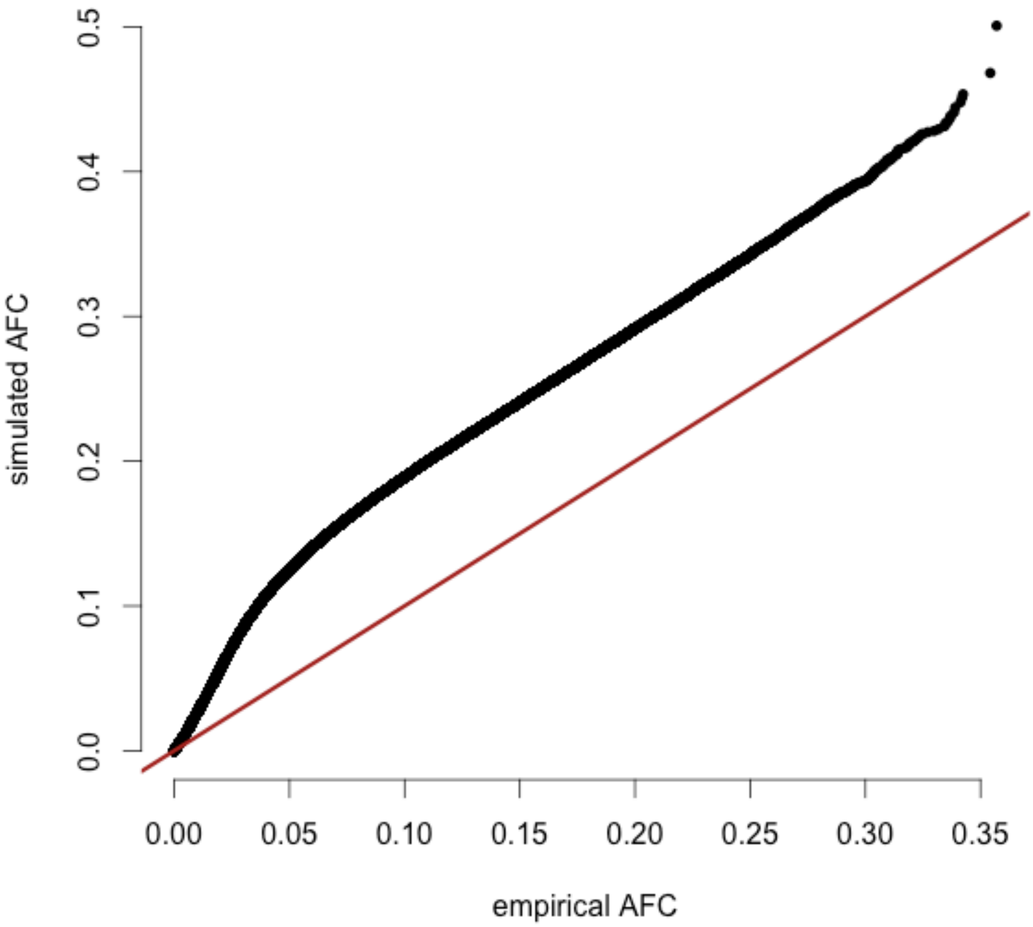

Figure S1. Quantile-Quantile plot of simulated versus empirical allele frequency changes (AFC) for *D. melanogaster*. Simulations assume four copies of each chromosome segregating in mated females (see main text for details). The identity line is displayed in red.

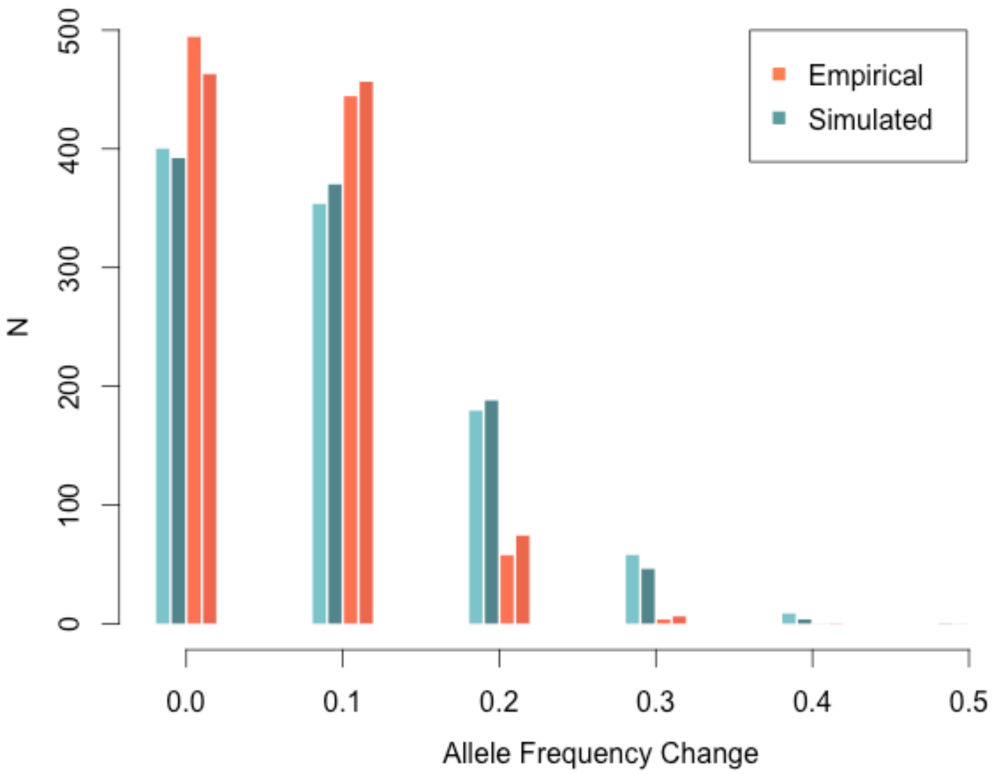

Figure S2. Distribution of simulated and empirical allele frequency changes (AFC) for *D. simulans*. Counts were normalized between simulated and empirical datasets. Each bar represents a replicate.
